# Elastocapillarity-driven 2D nano-switches enable zeptoliter-scale liquid encapsulation

**DOI:** 10.1038/s41467-023-44200-3

**Published:** 2024-01-02

**Authors:** Nathan Ronceray, Massimo Spina, Vanessa Hui Yin Chou, Chwee Teck Lim, Andre K. Geim, Slaven Garaj

**Affiliations:** 1 Department of Physics, National University of Singapore, Singapore, 117551 Singapore; 2 Centre for Advanced 2D Materials, National University of Singapore, Singapore, 117542 Singapore; 3 Department of Biomedical Engineering, National University of Singapore, Singapore, 117583 Singapore; 4 Institute for Health Innovation and Technology (iHealthtech), National University of Singapore, Singapore, 119276 Singapore; 5 Mechanobiology Institute, National University of Singapore, Singapore, 117411 Singapore; 6 National Graphene Institute, University of Manchester, Manchester, M13 9PL UK; 7 Department of Material Science Engineering, National University of Singapore, Singapore, 117575 Singapore

**Keywords:** Two-dimensional materials, Nanofluidics

## Abstract

Biological nanostructures change their shape and function in response to external stimuli, and significant efforts have been made to design artificial biomimicking devices operating on similar principles. In this work we demonstrate a programmable nanofluidic switch, driven by elastocapillarity, and based on nanochannels built from layered two-dimensional nanomaterials possessing atomically smooth surfaces and exceptional mechanical properties. We explore operational modes of the nanoswitch and develop a theoretical framework to explain the phenomenon. By predicting the switching-reversibility phase diagram—based on material, interfacial and wetting properties, as well as the geometry of the nanofluidic circuit—we rationally design switchable nano-capsules capable of enclosing zeptoliter volumes of liquid, as small as the volumes enclosed in viruses. The nanoswitch will find useful application as an active element in integrated nanofluidic circuitry and could be used to explore nanoconfined chemistry and biochemistry, or be incorporated into shape-programmable materials.

## Abstract (web summary)

In this work, authors demonstrate programmable nanostructures using two-dimensional materials for nanoscale liquid manipulation. The nanoswitches and capsules can hold zeptoliter liquid volumes, enabling active nanofluidics circuits and confined reactors.

## Introduction

In the natural world, living structures exhibit a programmable response to external stimuli—such as mechanical, chemical, or electrical—and adapt their properties, shape, and function to match the changing environment^1^. Intelligent materials and devices^2^, including emerging nanofluidic devices^3,4^, are foreseen to mimic such functionalities of living matter, combining sensing, actuation, and computation. Reversible shape-switching^5–7^ based on chemical and physical signals lead to design of artificial muscles^8^ and microscopic robots^9^. The contemporary programmable structures^10^ operate at scales above 10 µm due to mechanical limitations of solid-state thin films and elastomers that drive the switching process^8^. Here we developed a nanoscale switch operating at the 10-nm characteristic scale, based on nanochannels in two-dimensional materials, undergoing controlled elastocapillary transitions. We rationalized the switching process based on geometry, material, and liquid properties. This insight allowed us to rationally design the device geometries for desired functionalities and we demonstrated operation of switchable nanocontainers capable of enclosing zeptoliter volumes. Our mechanically switchable nanofluidic systems could further scale down on-device chemistry beyond impressive microfluidic technologies^11–13^ and could become an active element in adaptable nanofluidic circuitry.

Capillarity defines liquid–gas interfaces, drives the wetting of porous media^14^ and, not least, allows trees to pull water up their xylems over tens of meters^15^. In nanometer-sized pores, capillary pressures of several hundreds of bars build up^16^, and such dramatic pressures can deform the confining medium through a phenomenon known as elastocapillarity^17,18^. In semiconductor foundries, wetting and drying steps introduce a risk of elastocapillary damage and can trigger unwanted adhesion, thus hindering the miniaturization of electronic systems^19,20^.

**Fig. 1:**
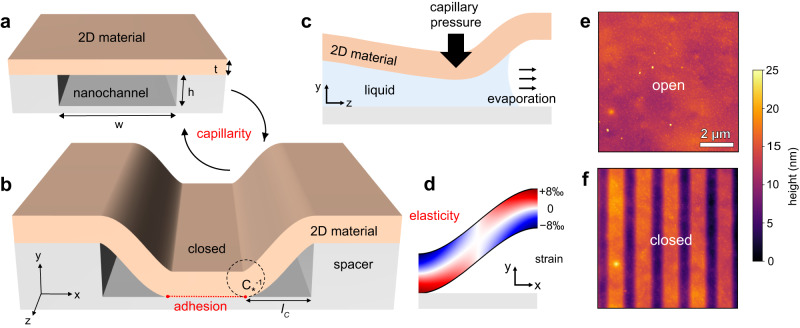
Elastocapillarity-switched adhesion in nanochannels. **a** Sketch of a nanochannel device in the stiff configuration. **b** Nanochannel in its caved-in state, where the top flake adheres to the substrate. ${C}_{\star }$ is the peeling curvature parameter and ${C}_{\star }^{-1}$ thus corresponds to the radius of curvature (dashed circle). **c** Upon drying the channel, capillary pressure triggers the caving-in of the top wall. **d** Calculated strain map in the bent cross-section using blue for compressive and red for tensile strain. The strain color scale refers to device D1, presented in (**e**, **f**) AFM (Atomic Force Microscopy) height maps of hBN/Gr/SiO_2_ nanochannels in their stiff and caved-in configurations, respectively.

In this work, we designed 2D nanochannels in which the action of elastocapillarity is reversible, and we employ it as a mechanical switching mechanism. We used two-dimensional layered materials (graphene, hBN, or WS_2_) as building blocks, as they can achieve nanometer-sized features while displaying atomically smooth interfaces and outstanding mechanical properties^21^. The nanochannels are designed by stacking several layered 2D crystals^22^ on top of the SiO_2_/Si substrate: (a) the bottom wall of the channel is defined by either a 2D crystal or base SiO_2_ substrate; (b) another 2D crystal is patterned into stripes to define the sidewalls of the nanochannel; (c) and the final 2D crystal, with atomically controllable thickness, defines the top wall and operates as an actuator. This nanoscale capillarity-actuated structure—*nanoswitch*—is designed by tuning the width and height of the channels and the thickness of the top wall (Fig. 1).

Two-dimensional nanochannels with sub-nanometer height but very stiff walls have been used to explore water^22^ and ion flow^23^ in the regime of extreme confinement. Ängstrom-scale “sagging” in such stiff channels has been used to monitor the presence of water in channels^24^ and explore the onset of capillary condensation^25^. These deformations were attributed to van der Waals interactions with the channel sidewalls, which is a distinct mechanism from the phenomenon at play in this work. Instead, we designed the 2D nanochannels to operate in different physical regime, where the channel walls could cave in under the capillary forces. To achieve the nanoswitch, the channels must satisfy two criteria: capillary forces must be strong enough to bend the top channel wall; and the adhesion between the top and bottom wall should be countered by the top layer stiffness to allow for reversible channel opening upon wetting. The interplay between elastocapillarity and adhesion determined the 10-nm scale motion of the top channel wall under the action of capillary pressures exceeding 100 bar, realizing new nanoscale machines which react not to traditional chemical^26^ or electromechanical stimuli^27^ but to liquid surface tension.

## Results and discussion

### Design and operation of the nanoswitches

We designed nanochannels using van der Waals assembly of mechanically exfoliated crystals of graphite (Gr), hexagonal boron nitride (hBN) or tungsten disulphide (WS_2_) transferred onto a silicon substrate with a thermal oxide layer (SiO_2_). The graphite crystal (thickness: $h\sim 10-20\,{{\mbox{nm}}}$) was patterned into stripes using electron beam lithography and reactive ion etching to define *spacers*. The channel width ($w=400-1000\,{{\mbox{nm}}}$) and the top crystal thickness ($t=10-40\,{{\mbox{nm}}}$) defined the top channel wall flexibility. For parallel channels, the pitch did not play any role in the observed phenomena, so we chose it large enough $p=1-2\,{\upmu }{{{{{\rm{m}}}}}}$, to allow diffraction-limited imaging of individual channels. Details on the fabrication procedure can be found in “Methods”. The channel geometry is illustrated in Fig. 1a.

The channels were filled with various liquids and subsequently, the devices were blow-dried with nitrogen. The drying process could lead to the caving-in of the top wall onto the substrate, as sketched in Fig. 1b. Similar observations at larger scales were reported in micromachined channels^19,28^ and in high-aspect-ratio nanopillars^29,30^. They are attributed to a twofold process: the nanostructure is deformed under the capillary pressure that builds up at the liquid–air interface^31^ (Fig. 1c), and adhesion maintains the deformation after drying.

**Fig. 2:**
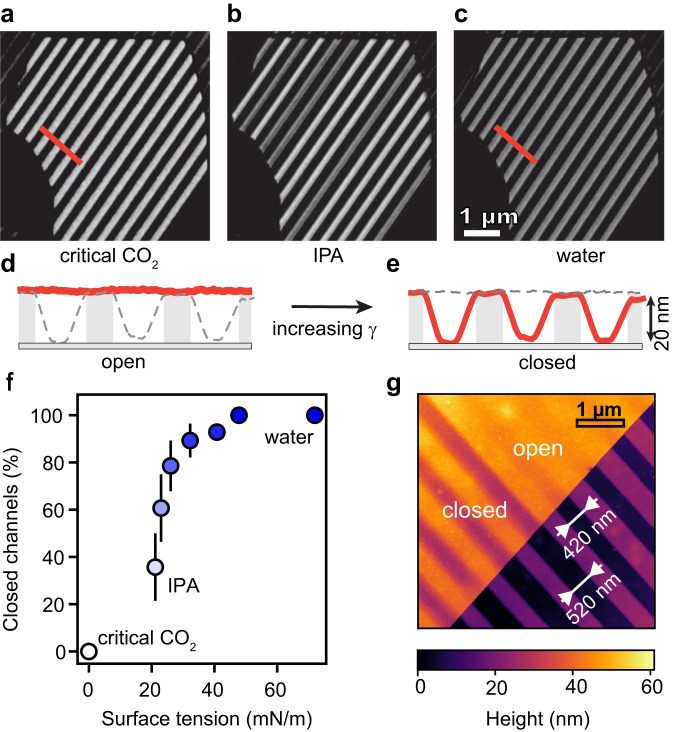
Visualizing and quantifying the nanoswitch optically and with Atomic Force Microscopy (AFM). **a**–**c** Optical micrographs of nanochannel device D2 after drying with different liquids. The bright and dark gray lines correspond to open or collapsed nanochannels, respectively. **d**, **e** Correlating the optical image to AFM profiles of channels after varying drying conditions. **f** Caved-in channels in the thick top region were optically counted as in (**b**) after drying with liquids of different surface tensions. IPA/water mixtures were used to interpolate the surface tension from 21 to 72 mN/m. Error bars correspond to standard deviations over three wetting/drying measurements. **g** Visualizing the width threshold for caving under capillary pressure: AFM map of hBN/Gr/SiO_2_ device D3 with channels of two different widths, switched by water capillarity stimulus. The bottom-right half (dark) of the image presents open spacers, with $h=17\,{{{{{\rm{nm}}}}}}$ high graphene sidewalls. Top-left (bright) part shows the same spacers topped with $t=22\,{{{{{\rm{nm}}}}}}$ thick hBN top wall, defining the nanochannels—the upper set of channels is open, while the lower is collapsed.

In Fig. 1d, we present a strain map in the channel cross-section calculated for our devices with a model introduced below, leading to both compressive and tensile strain in the order of a few percent. The changes between the stiff and caved-in configuration are visualized using atomic force microscopy (AFM) in tapping mode (Fig. 1e, f), here for device D1 using a hBN top crystal, graphite spacers, and a silicon oxide substrate (abbreviated as hBN/Gr/SiO_2_). The choice of hBN as a top wall material was motivated by its transparency, which allowed imaging of the nanoswitch under a standard wide-field optical microscope. We show in Fig. 2a–c that the mechanical changes in the crystal correspond to optical contrast (for thick enough spacers). As presented in Supplementary Movie 1, high-speed dark-field imaging enabled capturing these mechanical deformations as they happened.

We demonstrated the operation of the switch in device D2 (Fig. 2), with top channel walls made from hBN of thickness of $t=34\,{{\mbox{nm}}}$, exhibiting a good optical contrast between flat on-state and caved-in off-state. Upon critical point drying (that is, ethanol is first replaced by liquid CO_2_, which is driven beyond its critical point), all channels are open (Fig. 2a). Upon isopropanol (IPA) drying, we find that a portion of channels is in the off-state (dark) and the rest in on-state (bright); see Fig. 2b. Filling the channels with water followed by drying causes all channels cave in and appear dark (Fig. 2c). The observed optical contrast can be directly correlated to the topography measured by AFM: Fig. 2d, e shows height profiles along the colored lines in Fig. 2a–c.

Replacing the liquid leads to different surface tension, and thus the magnitude of the capillary force during channel drying. During CO_2_ critical point drying, there is no liquid–gas interface, and therefore no capillary pressure builds up. Water has a high surface tension $\gamma=72\,{{{{{\rm{mN}}}}}}/{{{{{\rm{m}}}}}}$ and IPA has a lower value ($\gamma=21\,{{\mbox{mN}}}/{{\mbox{m}}}$), which is why IPA removal leaves some channels open. We used water-IPA mixtures to control the magnitude of the surface tension^32^ and counted closed channels as a function of the surface tension. This defines the switch-stimulus curve in Fig. 2f.

Closed channels remained closed for months, as long as they were dry, but they relaxed to their open position upon rewetting. We were able to *reversibly* switch from the on- to the off-state, which could be repeated many times (Supplementary Figs. S8 and S9). The only limitation that could arise from repeated drying is cumulative contaminations of the wall surfaces with impurities dissolved in the liquid, which could change the adhesion energy between the top crystal and the substrate. For this reason, we excluded acetone in our study, as it leaves residues. We could easily regenerate acetone-contaminated devices by exposing it to hot acetone baths, followed by IPA rinsing.

We identified two limiting factors for the successful elastocapillarity-induced nanoswitch. First, for the top channel wall to cave in under the capillary pressure, it must be flexible enough, which we call the *caving-in criterion*. Second, for closed channels to open upon rewetting, adhesion should not overcome wetting: this is the *reversibility criterion*. In both cases, the bending response of the top wall is crucial. Previously, the stretching and bending response of 2D materials have been studied with the so-called “blister test” whereby a uniform pressure is applied to a 2D material suspended over a circular hole^33^. The stiffness of *multilayer* van der Waals materials was characterized recently using the same approach, yielding bending stiffnesses ranging from ${10}^{-14}$ to ${10}^{-13}{{\mbox{J}}}$ for crystal thicknesses in the range of 10–20 nm^34^.

### Capillarity vs. stiffness: the caving-in criterion

The caving-in of the nanochannel is determined by the relative magnitudes of the elastic force coming from the stiffness of the top wall, and the capillarity force. The bending stiffness $D$ of a thin film of thickness $t$ is expected to scale as $D\sim {t}^{3}$, and consequently, channels with a thin top crystal cave in more easily than those with thicker top. Wider channels should cave in more easily under a given capillary pressure, and we observed this in Fig. 2g. Channels with $w=420\,{{\mbox{nm}}}$ remained open upon water drying, whereas slightly wider channels with $w=520\,{{{{{\rm{nm}}}}}}$ caved in (both channels have height $h=17\,{{\mbox{nm}}}$ and top wall thickness $t=22\,{{\mbox{nm}}}$).

We calculated the caving-in criterium using the instability arguments when balancing capillary pressure with the bending stiffness of the channel^31,35^:

1 $$\phi {w}^{4}{{{{{\mathcal{G}}}}}}{{{{{\mathcal{/}}}}}}D{h}^{2} \, > \, 1$$

Details of the calculations are given in Supplementary materials. Here, we introduced the wetting surface energy ${{{{{\mathcal{G}}}}}}{{{{{\mathcal{=}}}}}}\gamma (\cos {\theta }_{S}+\cos {\theta }_{T})$ of the channel^34^, given by the contact angle between the liquid and the substrate $({\theta }_{S})$ and top wall (${\theta }_{T}$). The prefactor $\phi$ is set by the boundary conditions^35^ and reads $\phi=1/96$. We used $\theta={65}^{\circ }$ for thick 2D material-water interfaces^36^, and $\theta={0}^{\circ }$ for organic solvents which exhibit perfect wetting and for silicon dioxide-water interface^37^. We determined the geometrical parameters using atomic force microscopy, while bending stiffnesses were obtained from ref. ^34^ and surface tension was taken from ref. ^32^. The continuum medium assumption and the Young–Laplace formula are known to be valid down to few-nm confinement^16,38^.

In Fig. 2g, we see two sets of nanochannels with different widths ${w}_{{{\mbox{off}}}}=520\,{{\mbox{nm}}}$ and ${w}_{{{\mbox{on}}}}=420\,{{\mbox{nm}}}$, while all the other parameters remain identical. Upon drying with water, wider channels cave in, while narrower ones remain open. Using $D={10}^{-13}$ J for 22 nm-thick hBN^34^, Eq. (2) predicts a width threshold for caving-in upon water removal of ${w}_{{{\mbox{th}}}}=485\,{{\mbox{nm}}}$, aligning well with the experimental results, ${{w}_{{{\mbox{on}}}} < {w}_{{{\mbox{th}}}} < w}_{{{\mbox{off}}}}$. As observed and predicted, the IPA surface tension is not high enough to trigger the caving-in for both sets of nanochannels (Supplementary Fig. S4).

### Adhesion vs. stiffness: collapsed channels

Accurately predicting the reversibility of caving-in—the capacity of collapsed channel to open upon wetting—is more complicated than the caving-in criterion. Interfacial properties of the adhering top and bottom walls may be affected by contaminant adsorption, chemical functionalization, and roughness of the surfaces. However, for 2D materials with smooth and reproducible surfaces, we could derive a reversibility criterion, as shown below, with sufficient accuracy to guide the design of our nanoscale switches.

To validate our method, we first derived the energy of dry, closed channels, and compared the calculated geometry of the collapsed channel with the experimental profile measured by AFM. The top crystal was modeled as an infinite plate with a bending stiffness $D$, adhering to a step-shaped substrate with adhesion energy $\varGamma$. As sketched in Fig. 1b, due to its stiffness, the top crystal does not perfectly conform to the step and is suspended over a length ${l}_{{{{{{\rm{c}}}}}}}$. Our model (details in Supporting Information) yields a simple polynomial shape for the bending height profiles:

2 $$H\,\left(x\right)=h\varLambda \,(x/{l}_{{{{{{\rm{c}}}}}}}),\, {{{{{\rm{where}}}}}} \, \varLambda \,\left(X\right)\equiv 2{X}^{3}-3{X}^{2}+1$$

**Fig. 3:**
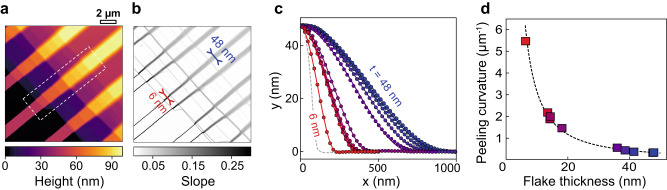
Adhesion/stiffness analysis of closed channels in device D4, obtained by exfoliating a terraced WS2 crystal on SiO2 spacers. **a** Atomic Force Microscopy height map of the collapsed nanochannels with varying top wall thickness $t$ ranging from 6 to 48 nm. **b** Slope extracted from the height map showing that the width of the transition zone increases with the crystal thickness. **c** Height profiles extracted along lines in the white dashed zone of (**a**) following the lines shown in (**b**). The dots are experimental points, and the solid black lines are the best fit to the polynomial profile obtained in our model. The top layer thickness was subtracted from the profiles. The *x* and *y* axis follow the convention defined in Fig. 1. **d** Peeling curvature ${C}_{\star }$ extracted from the profiles in (**c**), as a function of the crystal thickness. The dashed line is the best fit to a power law ${C}_{\star }\sim {t}^{-Q/2}$, yielding $Q=2.88\pm 0.08$.

To experimentally test our model, we designed a slightly modified version of the switch device using WS_2_ directly exfoliated onto a SiO_2_ substrate patterned with photolithography, ensuring contamination-free interfaces and precise bending profiles (device D4). A top crystal, with terraced thickness ranging from $t=6\,{{\mbox{nm}}}$ to $t=48\,{{\mbox{nm}}},$ was used to validate our model—presented as an AFM height map in Fig. 3a. The corresponding slope map in Fig. 3b is calculated as a derivative of the height map, offering evidence that the suspended length increases with increasing thickness, while the slope decreases. The experimental AFM profiles presented in Fig. 3c are very well fitted with Eq. (2).

The suspended length ${l}_{c}$ minimizes the sum of the elastic energy and the adhesion energy (per unit length) ${E}_{{{{{{\rm{elas}}}}}}}+{E}_{{{{{{\rm{adh}}}}}}}=12{D}{h}^{2}/{l}_{{{{{{\rm{c}}}}}}}^{3}-\varGamma (w-2{l}_{{{{{{\rm{c}}}}}}})$ and links it to the material parameters $D$ and $\varGamma$. As ${l}_{{{{{{\rm{c}}}}}}}$ depends on the height of the step as well as the material parameters, we define a geometry-independent parameter, the *peeling curvature* ${C}_{\star }=\sqrt{2\varGamma /D}=6h/{l}_{{{{{{\rm{c}}}}}}}^{2}$. It represents the minimum curvature needed to “peel off” the crystal from its substrate, which is obtained for $x=0$ and $x={l}_{{{{{{\rm{c}}}}}}}$, as illustrated in Fig. 1b. We can assume the adhesion energy to be thickness-independent for the thick flakes employed here^39,40^, therefore, the measured peeling curvature changes only with the bending stiffness of the material.

Figure 3d depicts the experimental variation of peeling curvature with the flake thickness. The peeling curvature is measured from the AFM scans as the curvature (approximated by the second derivative) at the contact line between the top and bottom walls, where the adhesion between the walls balances the bending stiffness of the top wall. The data is best fitted to a power law ${C}_{\star }\sim {t}^{-Q/2}$ (yielding a bending stiffness scaling $D\sim {t}^{Q}$, as shown in Supplementary Fig. S3) with $Q=2.88\pm 0.08$, which is close to the expected value of 3 for layered materials without slippage between planes^41^, and very close to recent results using blister tests on molybdenum disulphide^34^. Our model is validated by: (a) the near-perfect fit between our model and measured AFM profiles (Supplementary Fig. S1); and (b) its quantitative agreement of with published results obtained with different experimental techniques (Supplementary Fig. S2).

Our method of stiffness/adhesion measurements has additional applications: it can be used to measure the adhesion energy between different van der Waals materials and substrates if the bending stiffness is independently determined by other techniques. Using stiffness values $D$ for different crystal thicknesses from the literature^34^, we obtain $\varGamma=82\,{{{{{\rm{mJ}}}}}}/{{{{{{\rm{m}}}}}}}^{2}$ for WS_2_–SiO_2_ adhesion energy from the peeling curvature dependence on the thickness in the Fig. 3d (we assumed that WS_2_ has a similar bending stiffness as MoS_2_). Previous studies of adhesion typically use spontaneously occurring blisters^42,43^, and thus rely on assumptions on the poorly characterized content of the blisters^44^. Our method could be used for a variety of interacting materials, addressing the discrepancies in the literature regarding 2D material adhesion energies^45,46^.

### Adhesion vs. stiffness vs. capillarity: rewetting and the reversibility criterion

The rewetting process starts with dry, closed channel with a strongly adhering region in the center, and two narrow side-capillaries defined by the side wall and bent portion of the top wall, and the base of width ${l}_{{{{{{\rm{c}}}}}}}$ (see Fig. 1b). When introducing the liquid, it creeps into the side-capillaries and enlarges their base from the dry value ${l}_{{{{{{\rm{c}}}}}}}$ to the new value ${l}_{{{\mbox{wet}}}}$ due to the added contribution of the wetting interaction to the overall energy of the system (see Supplementary Fig. S5).

To find the new equilibrium value of the suspended length ${l}_{{{\mbox{wet}}}}$, we minimized the total energy ${E}_{{{{{{\rm{elas}}}}}}}+{E}_{{{{{{\rm{adh}}}}}}}+{E}_{{{{{{\rm{wet}}}}}}}$, where ${E}_{{{{{{\rm{elas}}}}}}}+{E}_{{{{{{\rm{adh}}}}}}}$ is calculated above, and ${E}_{{{{{{\rm{wet}}}}}}}=\gamma l\left(\cos {\theta }_{T}+\cos {\theta }_{S}\right)$ (see detailed derivation in Supporting Information). We achieve the full wetting when the base suspended length in the wet state becomes larger that the half width of the channel, ${l}_{{{{{{\rm{wet}}}}}}} > w/2$. This is the reversibility criterion, when wetting completely releases the top wall and it corresponds to the following inequality: $\frac{{w}^{2}{C}_{\star }}{h}{\left(1-\frac{{{{{{\mathcal{G}}}}}}}{\varGamma }\right)}^{1/2} \, < \, 24$.

### Nanoswitching phase diagram

The elastocapillary-induced nanoswitch is achieved by choosing the right set of geometric parameters $\{w,h,t\}$ and materials $\{\varGamma,D,{{{{{\mathcal{G}}}}}}\}$, yielding at least five independent parameters. Introducing the dimensionless parameters ${\alpha \equiv w}^{2}{C}_{\star }/h$ and $g\equiv {{{{{\mathcal{G}}}}}}{{{{{\mathcal{/}}}}}}\varGamma$, we reduced the complexity of this system to a two-dimensional space, and the universal *nanoswitch criterion* now reads:

3 $$8\sqrt{3}\,{g}^{-1/2} \, < \, \alpha \, < \, 24{\left(1-g\right)}^{-1/2}$$

where the left inequality corresponds to the caving-in criterion and the right inequality to the reversibility criterion. The value of $\alpha$ is defined by the channel geometry and the materials choice, while $g$ is defined by liquid and material interface properties.

**Fig. 4:**
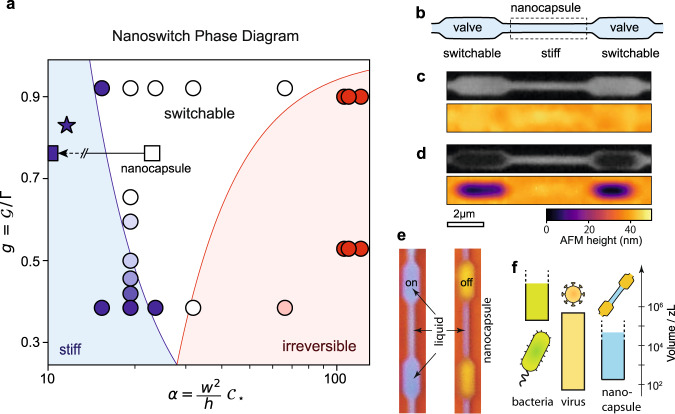
Generalization and application of the nanoswitch. **a** Universal nanoswitch phase diagram. The dimensionless parameter $\alpha={w}^{2}{C}_{\star }/h$ defines the channel geometry-dependent flexibility, and $g={{{{{\mathcal{G}}}}}}{{{{{\mathcal{/}}}}}}\varGamma$ defines the material wettability/adhesion ratio. Markers represent experiments for different geometry, solvents, and materials. Different regions in the parameter space are color-coded. For low $\alpha$, channels do not cave in upon liquid removal; this is the stiff region (blue). For high $\alpha$ and low $g$, the channels irreversibly collapse on the substrate (red). The remaining space is the switchable region, where channels undergo on/off transitions during wetting/drying (white). The star denotes the mechanical stability upper limit empirically established for atomically thin 2D nanoslits^22^. Squares denote parts of the nanocapsule: the open square is the switch part, and the blue square is for the container (off-scale, real value for container is $g \, \approx \, 4$). **b** A sketch of a self-sealing nanocapsule, consisting of a narrow stiff nanocontainer, delimited by two switchable valves. **c** Implementation of the nanocapsule, visualized in open configuration by optical microscopy (top) and AFM (bottom). **d** The same nanocapsule in closed state, where IPA removal switches off the valves, sealing the nanocontainer with zeptoliter volume. **e** Color-scale optical image of an open nanocapsule filled with liquid (blue) with open nanoswitch gates (left). The same nanocapsule with closed off nanoswitch gates, sealing in the ~100 zL of liquid inside (right). **f** Comparison of the range of volumes encapsulated inside bacteria, viruses and different nano-capsules implemented in this work.

The $\alpha -g$ phase diagram in Fig. 4a summarizes the predictions of the nanoswitch criterion and compares them to the experimental results. The blue region corresponds to “unbendable” channels which are too stiff to cave in under the liquid capillary pressure (for a given liquid), failing to pass the caving-in criterion. The red region corresponds to channels which fail to pass the reversibility criterion and remain closed even after rewetting. The white region passes both criteria and allows for reversible switching.

Each experimental data point in Fig. 4a represents a drying experiment followed by a check of the device configuration. White circles represent switchable channels that close upon drying and open upon rewetting. Blue markers represent stiff channels that remain open all the time, and red markers represent channels that close irreversibly upon drying. The scale of color for the remaining markers represents the success rate for closing/irreversible collapse when multiple channels are involved, ranging from 0% (blue/red) to 100% (white), as in Fig. 2. Near the transition line, there is a distribution of switchability of nominally equivalent channels, due to some channel-to-channel variability of the geometric and adhesion parameters. However, when moving away from this transition line, results are obtained deterministically.

Our experimental data shows good agreement with the predicted $\alpha -g$ phase diagram, demonstrating its usability in designing functional nanofluidic circuits (see below). The light red circle in the bottom-right part of the diagram only partially follows the expected irreversibility criterion, which might be due to the ill-defined interface between the bottom (SiO_2_) and top flake (WS_2_), possibly affected by contaminations of surface roughness. For this configuration, only 1 out of 5 nanochannels is irreversible. However, overestimating irreversibility is not a significant issue when designing switches, since we are mostly concerned to be either in the stiff or the switchable regime.

Van der Waals nanoslits with heights set by the thickness of monolayer/bilayer graphene spacers have been used to explore a variety of nanofluidic properties^4,22,23,47^. The design constraints for such structures have been empirically established: for 0.7 nm-high, 130 nm-wide channels to remain stiff, one must use top crystals thicker than 50 nm^22^. Our analysis supports this observation, denoted as a star on Fig. 4a, and those parameters are found within stiff region. Our results suggest that the geometrical criterion could be relaxed even further when performing nanofluidic experiments—even if a nanochannel is in the “switchable” region, it could readily recover its shape upon wetting, making them suitable for liquid and ion transport experiments despite caving-in in the dry state.

### Nanocapsule with zeptoliter volume

The nanoswitch could be used in two ways: (1) it could react to changes in the fluid medium through its surface tension-sensitive shape response or (2) nanofluidic circuits could have switchable parts with geometry-sensitive response that could modify their structure and function. To demonstrate the latter, we used the $\alpha -g$ phase diagram to design a *nanocapsule*—an active nanofluidic system consisting of switchable gates delimiting a stiff narrower container (Fig. 4b). The gates are nanochannels (width ${w}_{G}=2\,{{\upmu }}{{\mbox{m}}}$) that switch off due to capillarity, while the container is a narrower section of the channel (width ${w}_{C}=600\,\,{{{{{\rm{nm}}}}}}$) that does not cave in. The height of the channels is $h=37\,{{\mbox{nm}}}$, the top wall is made of hBN and the bottom from graphene. The nanocapsule parameters are presented in the $\alpha -g$ phase diagram in Fig. 4a as squares: the switch has $\alpha \, \approx \, 23$ (switchable region) and the stiff container has $\alpha \, \approx \, 4$ (out of scale).

In Fig. 4c, the nanocapsule has open gates, as shown in optical (top) and AFM (bottom) image. In Fig. 4d, the gates were closed by elastocapillarity, delimiting a container with a volume in range of ~100 zL, which could realistically be scaled further down. Figure 4f compares the range of enclosed liquid volumes accessible with nano-capsules with volumes in the range of bacteria and viruses. A time sequence of the drying process is provided in Supplementary Fig. S6, showing the successful trapping of liquid for ~10 s. This trapping time, sufficient to observe fast confined phenomena, could be improved significantly through optimizing the leakage between the walls and maximizing the gate length. In the case where electrolytes and solute are present, their concentration in a nanocapsule could diverge from the bulk concentration due to the drying dynamics, possibly leading to change in local surface tension and precipitation. To avoid any possible adverse effect, the initial and final electrolyte concentration should be monitored, while relatively short sections of the switchable channels, compared to the capsule, could be used to limit the effect. In the case of binary solutions, such as series of IPA-water mixtures used in Fig. 2, we do not observe any distortion in the drying behavior that might indicate a noticeable change in the local concentrations.

Slit-shaped 2D van der Waals nanochannels are a unique platform for the study of a wide range of properties of confined fluids^3,4^. In this work, we have demonstrated the switching behavior of 2D nanochannels, driven by elastocapillarity, and developed a theoretical framework to explain the phenomenon and rationally design such nanoswitches. Switchable nanochannels should be designed in two steps: (1) choosing the top wall materials which set $\varGamma$ and ${C}_{\star }$ and (2) identifying the right spacer dimension ratio ${w}^{2}/h$.

As we employ stiff but strong van der Waals materials as nanochannel walls, we could design nanofluidic switches operating at the single-digit nanometer scale. Previously, capillarity-switched adhesion was achieved in millimeter-sized channels using electroswitching^48^, but these structures rely on soft elastomers that limit their miniaturization below the micron scale.

We also designed a functional nanocapsule consisting of two switchable gates enclosing a stiff container, which allowed us to confine a zeptoliter-scale volume by using elastocapillarity to switch off the gates. These nano-capsules provide an exciting opportunity to explore phenomena at extremely small volumes, such as those found in viruses or below. As the walls of the nano-capsules are made of atomically smooth van der Waals materials with controllable surface chemistry, they could serve as a versatile platform for investigating nanoconfined biochemistry and chemistry^49^—provided a precise control of the local solution concentration is maintained. Furthermore, the nano-capsules could be incorporated into shape-programmable intelligent materials or used as active elements in integrated nanofluidic circuitry.

## Methods

### Fabrication

The patterning of spacers was done using electron beam lithography (JEOL JBX-6300FS) or photolithography (LW45B). The pattern was written on a polymer mask (PMMA and S1805 for e-beam or photolithography, respectively) and transferred to the substrate using deep reactive ion etching (Oxford Plasma Pro Cobra 100) with O_2_ and SF_6_ and CHF_3_ gases for etching graphite and SiO_2_, respectively.

### Imaging

The optical contrast of the images, resulting from thin-film interference, was a function of the spacer thickness as well as that of other layers. Rather than identifying the wavelength of optimal contrast, we recorded RGB images and used the channel with the most contrast. Optical images were acquired with an Olympus CX microscope in bright-field mode with a ×100 objective using white LED illumination. For device D2, the micrographs present only the red component of the color camera, as the green and blue components were found not to contribute significantly to the optical contrast. Conversely, in device D3, the green channel had the most contrast, as shown in Supplementary Fig. S7. The selected channel of the RGB images underwent a linear contrast enhancement.

Atomic force microscopy was performed with a Bruker FastScan AFM in tapping mode. Supercritical CO_2_ drying was performed using Leica EM CPD300.

### Wetting/drying experiments

Channels were immersed in isopropanol, which was solvent exchanged by transferring the wet chip to a beaker containing the desired liquid. We then let channels dry under the optical microscope or by directly blow-drying nitrogen gas. In Fig. 2a–f, closed channels were counted optically as being dark channels, between 0 and 15 in the region of interest. In cases where partial collapse was observed, a fraction corresponding to the closed length divided by the total length was assigned.

### Supplementary information

Supplementary Information

Description of Additional Supplementary Files

Supplementary Movie 1

### Source data

Source Data

## Data Availability

Source data are provided with this paper.
