# Supplementary material for: Elastocapillarity-driven 2D nano-switches enable zeptoliter-scale liquid encapsulation
Source: Nat Commun. 2024 Jan 2;15:185. doi: 10.1038/s41467-023-44200-3 (PMC10762047; doi:10.1038/s41467-023-44200-3)
Supplement: Supplementary file 1 — Supplementary Information [file 41467_2023_44200_MOESM1_ESM.pdf]

Supplementary Information

Elastocapillarity-driven 2D nano-switches enable zeptoliter-scale liquid encapsulation

Nathan Ronceray[1,2], Massimo Spina[1,2], Vanessa Hui Yin Chou[2], Chwee Teck Lim[3,4,5], Andre K. Geim[6], and Slaven Garaj[1,2,3,7*]

[1] Department of Physics, National University of Singapore, Singapore 117551

[2] Centre for Advanced 2D Materials, National University of Singapore, Singapore 117542

[3] Department of Biomedical Engineering, National University of Singapore, Singapore 117583

[4] Institute for Health Innovation and Technology (iHealthtech), National University of Singapore, Singapore 119276, Singapore

[5] Mechanobiology Institute, National University of Singapore, Singapore 117411, Singapore

[6] National Graphene Institute, University of Manchester, Manchester M13 9PL, United Kingdom

[7] Department of Material Science Engineering, National University of Singapore, Singapore 117575, Singapore

This file includes:

Detailed derivation of the bending profile model

Detailed derivation of Caving-in and Reversibility criterion

Figures S1-S9

Table S1: list of devices

Table S2: list of symbols

Detailed derivation of the bending profile model

We use a 1D continuum mechanics model adapted from a similar system[1] for the bending profile H(x) of a thin elastic flake conforming to a step-shaped substrate. The flake is clamped on the step substrate for $x < 0$, and is suspended over a length l_c at which it touches the substrate. The flake profile satisfies:

- $H''''(x) = 0$ for $0 < x < l_c$ as there is no load along the suspended part. We neglect long-range van der Waals interactions with respect to contact adhesion. Including them would lead to a correction of few % in the estimated parameters. Thus, the profile is a third-order polynomial $H(x) = a\,x^3 + b\,x^2 + c\,x + d$.
- The following natural boundary conditions:
  (i) $H(0) = h$,
  (ii) $H(l_c) = 0$: the flake follows the step.
  (iii) $H'(0) = H'(l_c) = 0$: the flake is *clamped* by adhesion.

The clamping boundary condition is supported by the experimental observation of zero slope at the contact points as well as previous measurements showing a high shear stress at the 2D material-silicon oxide interface[2]. In other words, the flake cannot slip on the SiO_2 substrate.

For simplicity, we set l_c and h as unit lengths and determine the scale-free bending profile $\Lambda(X)$, defined with $X := \frac{x}{l_c}$ and $\Lambda := \frac{H}{h}$. Solving the boundary conditions system yields the coefficients of the polynomial describing the flake profile:

$$\Lambda(X) = 2X^3 - 3X^2 + 1 = (X-1)^2\,(2X+1)$$

For now, we only used geometric boundary conditions. The measured value of l_c minimizes the total energy $E_{\mathrm{elas}} + E_{\mathrm{adh}}$.

The flake elastic energy per unit length is given by $E\mathrm{elas} = \frac{D}{2}\int_0^{l_c} C^2(x)dx$ where $C(x)$ is the local curvature of the top wall, defined by $C(x) = \frac{H''(x)}{\left(1+H'^2(x)\right)^{\frac{3}{2}}} \approx H''(x)$ as the slope remains small.

Approximation. Here we assumed that (narrow channel approximation):

$$H'^2(x) \ll 1$$

i.e., the maximal value $H'^2(x)|_{\max} = \frac{3}{8} h \sqrt{\frac{2\Gamma}{D}} \ll 1$, which is a good assumption in our case of small channel height h and larger stiffness of the top wall D. For our range of operational parameters this is always fulfilled, $h\sqrt{\frac{\Gamma}{D}} \sim 10^{-3} - 10^{-2}$. This assumption is further validated by the excellent comparison of experiments and theory in Fig 3c-d, Fig S1 and Fig S2.

Using the polynomial expression for the profile, we obtain $E_{\text{elas}} = \frac{6Dh^2}{l_c^3}$.

We can see in Figure S1 that all re-scaled profiles $Y = \frac{y}{h} = \Lambda(X) = \Lambda\left(\frac{x}{\ell_c}\right)$ collapse to the curve given by eq. 2, proving that we indeed derived the analytical solution (dashed line).

Detailed derivation of Caving-in and Reversibility

Caving-in criterion

The collapse of flexible channels under capillary forces has been investigated experimental and theoretically at larger scales[3,4]. We employ their calculations and revisit their phenomenological assumptions to suit our nanometer length-scales – leading to the caving-in criterion for our devices.

We introduce the deflection δ of the top wall due to the capillary pressure, and the relative deflection $\xi = \delta/h$. The deflection in the center along the width of the channel, and at the lowest point along the length of the channel will be given by balance of the strain energy and applied pressure:

$$\xi_{df} = \frac{\phi w^4}{4Dh} p_{df} \qquad \text{(S 1)}$$

Where $\phi = 1/96$, w is the width of the channel, h is the height of the channel, and p_{df} is the driving pressure that deforms the membrane, in our case the capillary pressure. The Young-Laplace formula for capillary pressure reads[5]:

$$p_{YL} = \frac{\mathcal{G}}{h(1-\xi)} \tag{S 2}$$

However, the capillary pressure is defined not necessary at the point of the highest deflection ξ_{df} , but it could be defined at some other deformation point $\xi_{cap} = \kappa\xi_{df}$. The κ is a phenomenological parameter, which considers a) bending of the channel along its length from the lowest towards the dry part of the channel; b) extension of the meniscus; and c) modification of the continuum physics at the length scales below several nanometers[6,7] (the latter could be important in our system). Such effect would lead to reduction of the driving pressure by factor κ compared to the maximum capillary pressure expected that the highest deflection point. Combining the equation (S 1) with the capillary pressure at position ξ_{cap}, we get expression:

$$\kappa\xi_{df}^2 - \xi_{df} + \frac{\phi\mathcal{G}w^4}{4Dh^2} = 0 \tag{S 3}$$

The above quadratic equation has no real solutions for:

$$\kappa\frac{\phi\mathcal{G}w^4}{Dh^2} > 1 \tag{S 4}$$

leading to the collapse of the channels in that case.

For the phenomenological parameter κ, van Honschoten *et al*[4] assumed the value of $\kappa = 1/2$ ("only a motivated estimate"), as it supported their experiments well. Anoop *et al*[3] followed their lead. This assumption fitted well the experimental results for their large channels.

In our case, we assume the parameter is $\kappa = 1$, due to much smaller length scales over which the meniscus develops in our devices, and the rigidity of or membranes. This value is supported by our experimental results and leads to the caving-in criterion (equation 1 in the main text).

To get a more precise estimate of κ, a numerical simulation should be performed. The resulting gain in precision might not have practical benefits, as it would be below the threshold of the device-to-device uncertainty and variability. The benefit of our Nanoswitch Phase Diagram lies in the fact that it could quickly offer good design decision when developing switching circuitry and could give insights on the influence of different parameters (solvents, materials, geometry) – without extensive simulations.

An alternative derivation of the caving-in criterion, leading to the same results, starts from the energy expression for channel undergoing bending of the top wall due to capillarity reads:

$$\Psi(\xi) \; = \; E_{\mathrm{elas}} - \; W_{\mathrm{cap}} = \frac{Kh^2}{2}\, \xi^2 \; + \; \mathcal{G}\log\left(\, 1 - \xi\right) \qquad \text{(S 5)}$$

Where we introduced the effective spring constant of the channel $K \; = \frac{4D}{\phi w^4}$ and the wetting surface energy $\mathcal{G} = \gamma\; (\cos\theta_S + \cos\theta_T)$ of the channel. The channels will collapse if the energy decreases ($\frac{\mathrm{d}\Psi}{\mathrm{d}\xi} < 0$) and equation (S 5) does not have local energy minima in the range $0 < \xi < 1$, that is, $\frac{\mathrm{d}\Psi}{\mathrm{d}\xi} < 0$ in this whole range.

$$\frac{\mathrm{d}}{\mathrm{d}\xi}\Psi(\xi) \; = \; \mathrm{Kh}^2\xi - \; \mathcal{G}\frac{1}{1-\xi} < 0 \qquad \text{(S 6)}$$

For the collapse channels, there should be no local minima in energy expression, hence the expression (S 6) should not have any real solutions. This leads to the requirement $\frac{4\mathcal{G}}{Kh^2} > 1$, and to the caving-in criterion in the main text (see equation 1, main text). This energy minimization requirement is equivalent to the requirement $\frac{\mathrm{d}\Psi}{\mathrm{d}\xi}\left(\xi = \frac{1}{2}\right) < 0$. See Figure S4 for the evolution of the energy with the relative deflection for different device parameters.

Reversibility criterion

The reversibility criterion can be obtained by considering an adhered, *wet* configuration (same as in Fig. 1b but with the gap under the suspended length l filled with liquid, see Fig. S5) sketched here. The profile $H(x)$ is still given by the polynomial expression but it is no longer parametrized by the dry, equilibrium value l_c but rather by a parameter l. Introduction of the liquid into the side capillary adds wetting energy to the calculations, leading to a new value of $l \geq l_C$.

The filling pathway in Figure S5 requires that the side capillary is first filled with water, which could be a comparatively slower process that drying. The value of the suspended length l in wet conditions is set by the channel wetting energy E_{wet} on top of the elastic energy and adhesion energy introduced in the main text. The equilibrium value of all these components is given by:

$$\frac{d}{dl}(E_{elas} + E_{adh} + E_{wet}) = 0 \qquad \text{(S 7)}$$

As in the main text, reasoning on the half-channel above, one obtains: $E_{\mathrm{elas}} + E_{\mathrm{adh}} = 6\frac{Dh^2}{l^3} - \Gamma\left(\frac{w}{2} - l\right)$, only the wetting energy remains to be computed. It is given by integrating the wetting energy $E_{wet} = -\gamma\left(l_{top}\cos\theta_T + l_{wet}\cos\theta_S\right)$, where θ_T is the contact angle between the liquid and the material of the top surface, and θ_S is the contact angle at the bottom surface, i.e. substrate.

Using contour integral, we calculate:

$$l_{top} = \int_0^l \sqrt{1 + H'(x)^2}\,dx \approx \int_0^l 1 + \frac{H'(x)^2}{2}\,dx = l + \frac{1}{2}\int_0^l H'(x)^2 dx$$

We replace $H(x)$ by its polynomial expression (equation 2, main text) and obtain:

$$l_{top} = l_{wet}\left(1 + \frac{3}{5}\left(\frac{h}{l_{wet}}\right)^2\right)$$

If we assume the same narrow channel approximation (equation S1), equivalent to $(h/l_{wet})^2 \ll 1$, we derive the wetting energy as:

$$E_{\mathrm{wet}} = \gamma l_{wet}(\cos\theta_T + \cos\theta_S)$$

The energy balance (equation S3) must now be solved to yield an equilibrium value l_{wet}. Two scenarios may occur:

- If $l_{wet} < \frac{w}{2}$ wetting does not release the walls (irreversible collapse)
- If $l_{wet} > \frac{w}{2}$ wetting releases the wall.

We therefore solve the equation $l_{eq} = \frac{w}{2}$ to obtain the boundary of the soft reversibility criterion. It reads

$$-\frac{18Dh^2}{l_{wet}^4} + \Gamma - \mathcal{G} = 0$$

Therefore, we obtain:

$$\left(\frac{h}{l_{wet}}\right)^2 = h\sqrt{\frac{\Gamma(1-g)}{18\,D}}$$

which can be rephrased as:

$$\alpha = 24\,(1-g)^{-\frac{1}{2}}$$

Figure S1

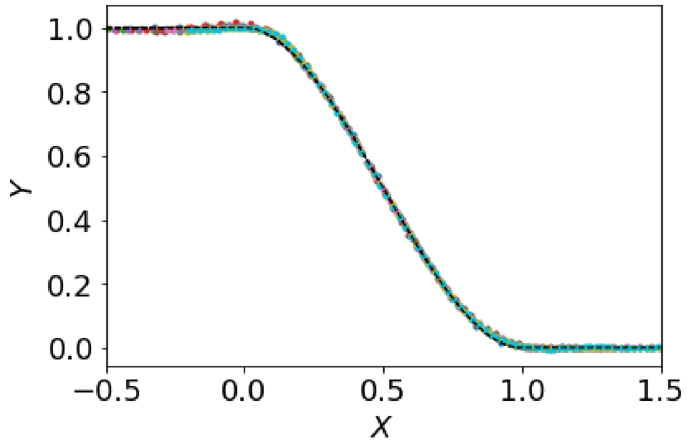

Figure S1: rescaled top wall bending profiles $Y = \frac{y}{h} = \Lambda(X) = \Lambda\left(\frac{x}{\ell_c}\right)$ for the data from Fig. 3, proving the validity of our analytical model.

Figure S2

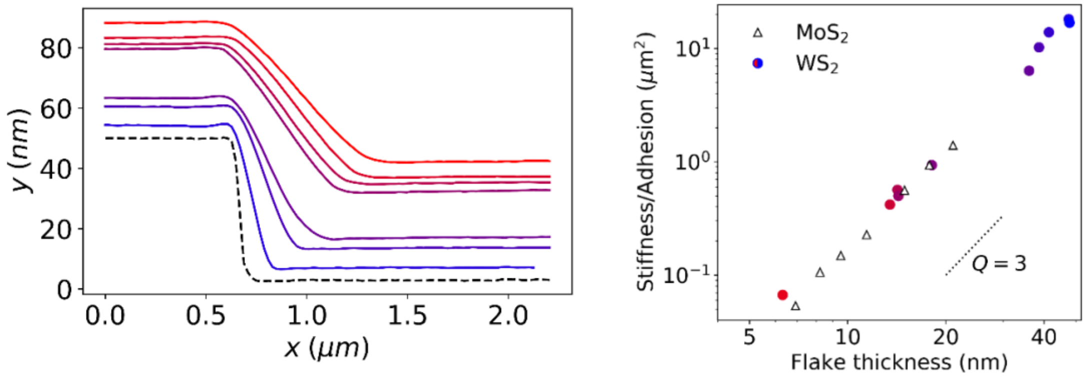

Figure S2: Left: Raw atomic force microscopy profiles without top layer thickness subtraction. Right: Stiffness/adhesion $\frac{D}{\Gamma}$ ratio as a function of the multilayer van der Waals material thickness. Colored dots correspond to data extracted from Fig. 3 in the main text, and white triangles correspond to values calculated from[8,9].

Figure S3

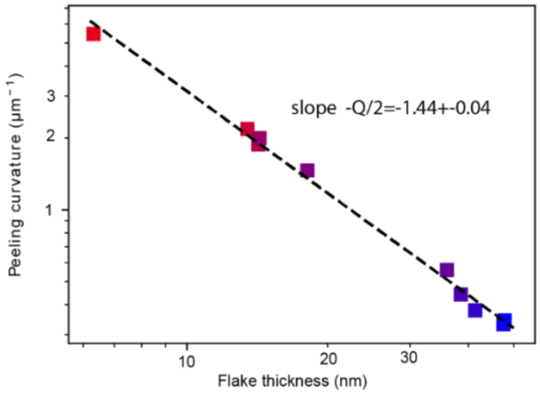

Fig. S3. Log-log plot of the relationship provided in **Figure 3**d. The dashed-line is the result of a least-square fitting to an affine function in log-log space, evidencing the power-law with an exponent very close to the expected -3/2.

Figure S4

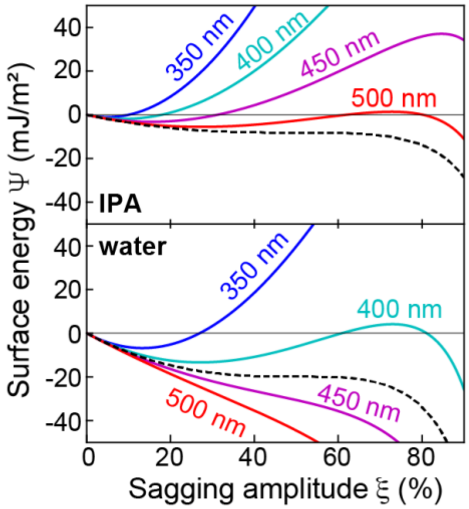

Figure S4: Predicted energy functional (per surface unit) for the capillary bending of the top wall as a function of the sagging amplitude $\xi = \delta/\text{h}$, calculated for different channel widths (labels), presented for IPA (top panel) and water (bottom panel). The dashed line predicts the width threshold separating open and closed channels for given liquids. For water, the width threshold is $w_{th} = 430$ nm, matching experimental observations.

Figure S5

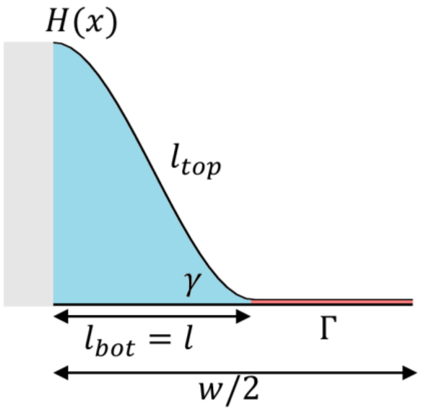

Figure S5: Sketch of the cross-section of a part of the collapsed nanochannel in the region close to the sidewall, with a narrow capillary between the sidewall and the collapsed top wall filled with liquid (blue). Adhered section of the top and the bottom wall is shown in red.

Figure S6

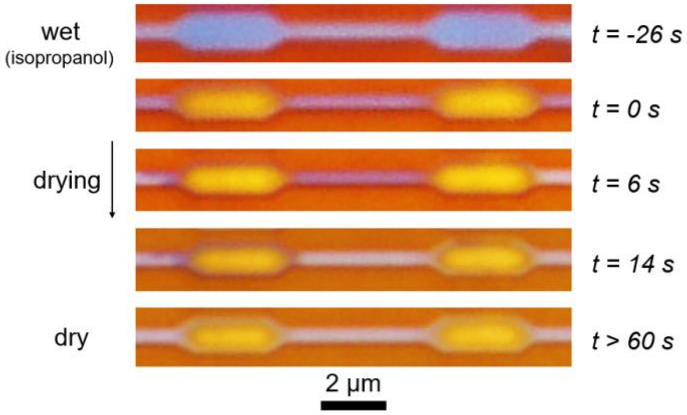

Fig S6: Liquid trapping in the nano-capsule, showing the successful trapping of liquid for ~10 seconds. The initial state (t=-26s) has the device covered with liquid. At t=0s, there is no liquid left on the device, and liquid remains trapped at t = 6s. At t=14s, the container is empty, with leftover liquid visible on the left side.

Figure S7

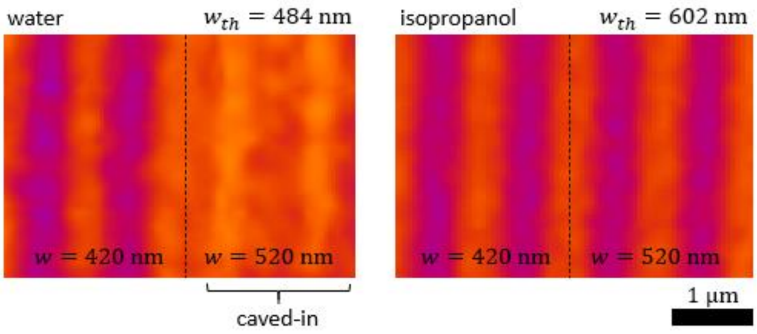

Fig. S7. Additional optical check corresponding to Fig. 2g. The green optical channel of the RGB camera was used for this device, providing suitable optical contrast. Note that in this geometry the darker parts correspond to caved-in channels.

Figure S8

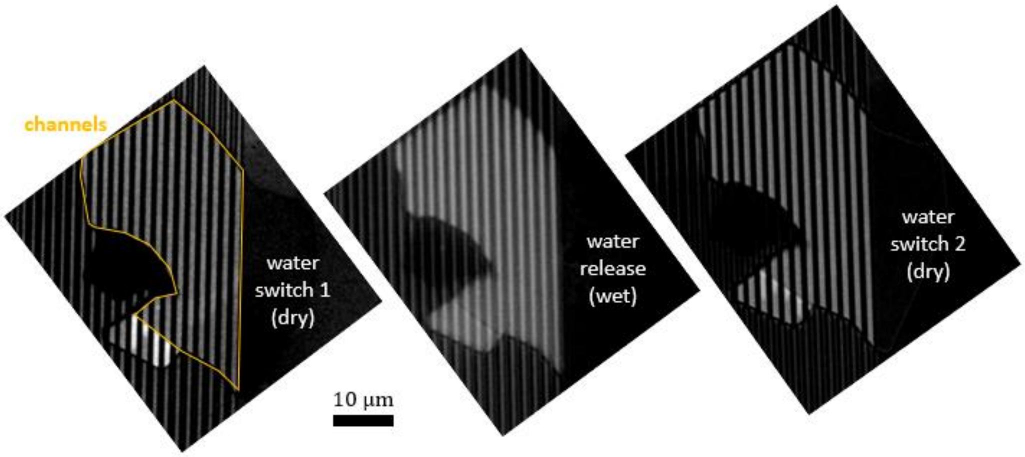

Fig. S8. Reversibility and repeatability of the switching phenomenon. The images above correspond to a full cycle of drying and wetting: we imaged device D2 after water switching (left), after which the device was imaged in water (center), and the drying was repeated (right). The bottom left region shows the expected optical contrast level for open channels in dry.

Figure S9

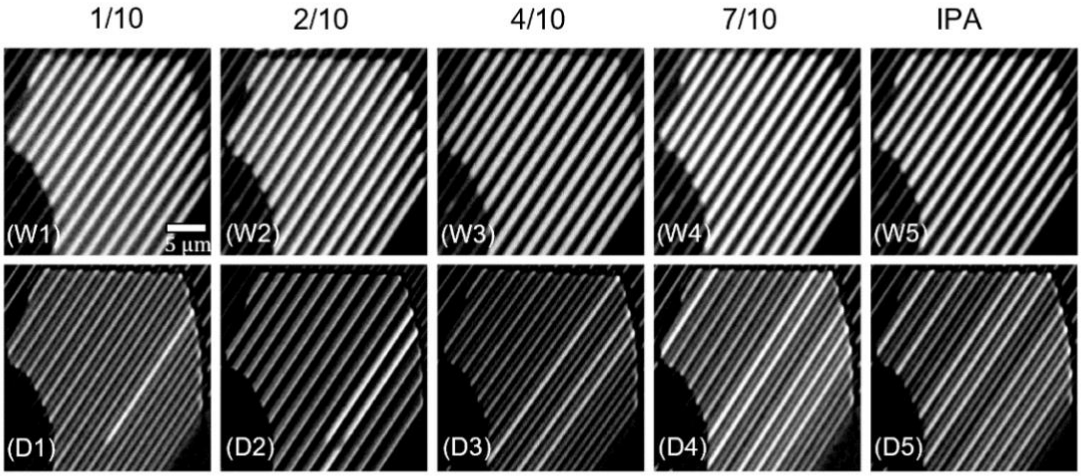

Fig. S9. Proof of the reversibility when changing the solvents (extended data from **Figure 2**). The top images, labelled (W1-5), correspond to wet channels which recovered from their previous collapse. Below, we show the outcome of the drying process with an IPA proportion corresponding to the fraction indicated above the images. This optical check confirms that channels are always released by re-wetting.

Supplementary Movie 1

Dark-field high-speed imaging of isopropanol removal in device D2. The sample was illuminated with white light using a mercury lamp (Nikon Intensilight C-HGFI) and the recording was performed at 500 fps (Photron FASTCAM SA3 monochrome), using a 100X air objective (NA=0.8) mounted on an upright microscope (Nikon Eclipse). Scale bar: 5 µm.

Table S1: list of devices

Device number	Materials (top/spacer/substrate)	Geometry: height / width / top thickness (nm)	Comments
D1	$hBN/Gr/SiO_2$	11 / 1000 / 44	None
D2	$hBN/Gr/SiO_2$	20 / 1100 / 12,34	Varying top thickness, and surface tension
D3	$hBN/Gr/SiO_2$	17 / 420,520 / 22	Varying channel width
D4	$WS_2/SiO_2/SiO_2$	48 / 2900 / 6-48	Varying top thickness, irreversible collapse
D5	hBN/Gr/Gr	28/600-2000/43	Nanocontainers

Table S2: list of symbols

Symbol	Meaning
Geometric	
h	Height of the channel
t	Thickness of the top channel wall
w	Width of the channel
p	Pitch between the channels
x	Coordinate along the width of the channel
y	Coordinate along the height of the channel
z	Coordinate along the length of the channel
H(x)	Height profile of the flexible top wall, along the width of the channel
δ	Maximum deflection of the top wall bent by capillarity
Material	
D	Stiffness of the top wall

Symbol	Meaning
Γ	Adhesion surface energy (per unit area) between top wall and the surface.
E_{elas}	Total elastic energy of bended top wall, per unit length of the channel
E_{adh}	Total adhesion energy between the caved-in top wall and the substrate, per unit length of the channel
l_c	Equilibrium value of the length over which the caved-in top wall is suspended (dry state)
l	Length over which the caved-in top wall is suspended, base of the "side-capillary"
l_{wet}	Equilibrium value of the length over which the caved-in top wall is suspended (wet state)
$C_\star$	Peeling curvature, minimum curvature needed to 'peel off' the crystal from its substrate. $C_\star = \sqrt{\frac{2\Gamma}{D}}$
Liquid and interfaces	
γ	Liquid surface tension
θ_S	Contact angle between liquid and the substrate (bottom surface)
θ_T	Contact angle between liquid and the top substrate
$\mathcal{G}$	Wetting surface energy
E_{wet}	Energy of wet caved-in channel, per unit length
Parametrization	
$\Lambda(X)$	Polynomial $\Lambda(X) \equiv 2X^3 - 3X^2 + 1$
α	Parametrization of geometric and material parameters, $\alpha \equiv w^2 C_\star / h$
g	Parametrization of liquid and material parameters, $g \equiv \frac{\mathcal{G}}{\Gamma}$

Supplementary References

1. Kim, H.-Y. & Mahadevan, L. Capillary rise between elastic sheets. *Journal of Fluid mechanics* **548**, 141–150 (2006).
2. Wang, G. *et al.* Measuring interlayer shear stress in bilayer graphene. *Physical Review Letters* **119**, 36101 (2017).

3. Anoop, R. & Sen, A. K. Capillary flow enhancement in rectangular polymer microchannels with a deformable wall. *Phys. Rev. E* **92**, 013024 (2015).
4. Van Honschoten, J. W., Escalante, M., Tas, N. R., Jansen, H. V. & Elwenspoek, M. Elastocapillary filling of deformable nanochannels. in *Journal of Applied Physics* vol. 101 094310 (American Institute of Physics AIP, 2007).
5. Israelachvili, J. N. *Intermolecular and surface forces*. (Academic press, 2015).
6. Van Honschoten, J. W., Brunets, N. & Tas, N. R. Capillarity at the nanoscale. *Chemical Society Reviews* **39**, 1096–1114 (2010).
7. Cheng, S. & Robbins, M. O. Nanocapillary adhesion between parallel plates. *Langmuir* **32**, 7788–7795 (2016).
8. Wang, G. *et al.* Bending of Multilayer van der Waals Materials. *Physical Review Letters* **123**, 116101 (2019).
9. Sanchez, D. A. *et al.* Mechanics of spontaneously formed nanoblisters trapped by transferred 2D crystals. *Proceedings of the National Academy of Sciences* **115**, 7884–7889 (2018).
