# Supplementary material for: Elastocapillarity-driven 2D nano-switches enable zeptoliter-scale liquid encapsulation
Source: Nat Commun. 2024 Jan 2;15:185. doi: 10.1038/s41467-023-44200-3 (PMC10762047; doi:10.1038/s41467-023-44200-3)
Supplement: Supplementary file 2 — Description of Additional Supplementary Files [file 41467_2023_44200_MOESM2_ESM.pdf]

Description of Additional Supplementary Files

File Name: Supplementary Movie 1
Description: Dark-field high-speed imaging of isopropanol removal in device D2. The sample was illuminated with white light using a mercury lamp (Nikon Intensilight C-HGFI) and the recording was performed at 500 fps (Photron FASTCAM SA3 monochrome), using a 100X air objective (NA=0.8) mounted on an upright microscope (Nikon Eclipse). Scale bar: 5 µm.
